# Increased incidence of Susac syndrome: a case series study

**DOI:** 10.1186/s12883-020-01892-0

**Published:** 2020-09-02

**Authors:** A. Wilf-Yarkoni, O. Elkayam, O. Aizenstein, Y. Oron, V. Furer, D. Zur, M. Goldstein, D. Barequet, H. Hallevi, A. Karni, Z. Habot-Wilner, K. Regev

**Affiliations:** 1 Neuro-Immunology Service and Department of Neurology Rabin Medical Center, 4941492 Petach Tikva, Israel; 2 Department of Rheumatology, Tel Aviv Sourasky Medical Center, Tel Aviv, Israel; 3 Sackler Faculty of Medicine, Tel Aviv University, Tel Aviv, Israel; 4 Neuroradiology unit, Department of Radiology, Tel Aviv Sourasky Medical Center, Tel Aviv, Israel; 5 Department of ENT, Tel Aviv Sourasky Medical Center, Tel Aviv, Israel; 6 Division of Ophthalmology, Tel Aviv Sourasky Medical Center, Tel Aviv, Israel; 7 Neuroimmunology and Multiple Sclerosis Unit of the Department of Neurology, Tel Aviv Sourasky Medical Center, Tel Aviv, Israel; 8 Sagol School of Neuroscience Tel Aviv University, Tel Aviv, Israel

**Keywords:** Susac syndrome, Treatment, cmv post infectious, Branch retinal artery occlusion

## Abstract

**Background:**

Susac syndrome (SuS) is a rare condition characterized by a clinical triad of sensorineural hearing loss, branch artery occlusion and encephalopathy. This study reports an increased incidence of SuS in Israel. We describe the clinical characteristics of these patients, diagnostic procedures and the use and subsequent outcomes of newly published treatment guidelines.

**Methods:**

This is a single center retrospective study. Patients who were diagnosed with SuS between July 2017 and August 2018 were enrolled in this study.

**Results:**

Seven patients were diagnosed with SuS according to the diagnostic criteria in a time period of 13 months. The annual incidence was recently evaluated in Austria to be 0.024/100000, therefore, our case series represent at least a 5.4- fold increase in the annual incidence of SuS expected in Israel and a 7-fold increase in the annual incidence expected in our medical center. Mean time from the onset of the symptoms to diagnosis was three weeks and follow-up time was twenty four months.

Recent exposure to cytomegalovirus was serologically evident in three patients and one patient had high titer of anti-streptolysin antibody. All patients underwent brain MRI, fluorescein angiography and audiometry.

All patients were treated according to the newly recommended guidelines. All patients achieved clinical and radiological stability.

**Conclusions:**

We report of an increased incidence of SuS in Israel. Infectious serological findings may imply a post infectious mechanism. The use of the recommended diagnostic procedures reduced the time to diagnosis. Newly published treatment guidelines led to favorable clinical outcomes.

## Background

Susac syndrome (SuS) is a rare immune-mediated occlusive microvascular disease. It is characterized by a typical clinical triad of encephalopathy, visual disturbances and hearing loss [1–5]. However, there is a great variability in clinical manifestations and the complete triad is present in less than 20% of patients at disease onset [4–9]. Treatment of SuS is particularly challenging, owning to its rarity, and the great variability in presentation, there are no randomized control trials to evaluate treatment strategies. Optimal outcome requires rapid and aggressive treatment, and acute treatments limited to glucocorticoids and/or IVIg appears to be insufficient to halt the progression of disease [10–15]. Recent treatment guidelines have been published based on disease severity [10].

The available data regarding the prevalence and incidence of SuS is limited. The annual incidence was recently evaluated in Austria to be 0.024/100000 [16]. The largest case series from Israel comprises 10 patients who were diagnosed over a period of 26 years [17].

In this retrospective case series, we report seven newly diagnosed cases of SuS in a single referral center over a time period of thirteen months. For a reference, from 2013 to 2016 only two patients were diagnosed with SuS in our institute. We hereby describe the clinical features, diagnostic procedures employed, treatment approach and outcome of this cohort.

## Methods

### Study design and data collection

This is a retrospective case series of patients treated at a single tertiary medical center. The study was approved by the Tel Aviv Sourasky Medical Center Institutional Review Board (Helsinki Committee). (0435-15-TLV).

All patients diagnosed with SuS, between July 2017 and August 2018 were included. The European Susac consortium criteria were used for diagnosis. According to these criteria, patients with involvement of all three main organs (brain, eye and ear) who fulfill the typical clinical triad were defined as definite SuS and patients with involvement of two main organs were defined as probable SuS (Supplementary Table 1) [18].

All patients were hospitalized in the neurological department in Tel Aviv Sourasky Medical Center and were examined by ophthalmologist and ear, nose and throat specialist. Data regarding the following parameters were collected: patient demographics, medical history, and medications. Clinical presentation including history, neurological assessment, treatment protocol, evidence of relapse and response to treatment were recorded. Detailed personal and occupational history was obtained together with possible environmental, toxic, chemical exposures according to the exposure survey.

All patients underwent extensive medical investigations to establish the diagnosis of SuS: blood tests and screening tests for infectious diseases; cerebrospinal fluid (CSF) analysis and diagnostic procedures including brain MRI, auditory evaluation, and Fluorescein angiogram (FA). Spectral-domain optical coherence tomography (OCT) was done in all cases since it is an emerging diagnostic tool in SuS [19, 20]. Macular OCT scans were evaluated for (1) areas of hyperreflective thickening of retinal nerve fiber layer to the outer plexiform layer which is indicative for tissue swelling due to acute branch retinal artery occlusion (BRAO), and (2) areas of thinning of these layers indicative for previous ischemic damage. Cerebral angiography was performed in two patients and brain biopsy was performed in one patient.

CNS involvement was characterized by both clinical and radiological evidence. Clinical symptoms included new cognitive impairment and/or behavioral changes and/or new focal neurological symptoms and/or new headache. MRI findings included typical findings on cranial MRI—hyperintense, multifocal, small round lesions; at least one of them in the corpus callosum (‘snowball’) in T2 (or FLAIR) weighted sequences [18]. Cervical spine MRI was done in one patient.

In order to evaluate treatment response, a special follow-up clinic was created with a multi-disciplinary team including a neurologist, rheumatologist and an ophthalmologist as well as continuing monitoring by MRI scans and FA. New or worsening of neurological, ocular or auditory symptoms and/or new lesions on brain MRI and/or evidence of new BRAO’s on FA determined a relapse. Clinical stability was defined as no evidence of clinical relapse, no new lesion on brain MRI and no evidence of BRAOs on FA study. Outcome measures included adverse events and clinical and para-clinical evidence of disease sequelae.

## Results

### Clinical characteristics

Table 1 summarizes the demographic data, signs and symptoms upon presentation. Seven patients (4 females, 3 males) were diagnosed with SuS. There is no available data on the incidence of SuS in Israel. Two patients were diagnosed with SuS in Tel-Aviv Medical Center from 2013 to 2016 (Supplementary figure 1). We calculated the expected number of patients per year in Israel, based on annual incidence evaluation in Austria of 0.024/100000 (age over 19), to be 1.3. Therefore, our case series represent at least a 5.4-fold increase in the annual incidence of SuS compared to a published registry and a 7-fold increase in the annual incidence expected in our medical center.

**Table 1:** Demographic and clinical presentation

	Time to diagnosis	Disease severity	Lumber Puncture			CNS involvment			Eye involvment		Ear involvment		Complete triad***	Serology	Non-neurological symptoms
			Opening Pressure (mm H2o)	WBC (cells/μl)	Protein (mg/dl)	Cognitive/ Psychiatric	Focal signs	Headache	S*	A-S**	S*	A-S**			
1.M/20	Two weeks	Severe	260	1	110	Cognitive + psychiatric	Aphasia and sensory disturbance	+		BL	BL		+	CMV IgM	GI symptoms
2.F/34	Two weeks	Moderate	80	14	125	Moderate encephalopathy	–	–		BL	UL+ Vertigo		+		
3.F/20	Three weeks	Mild	–	–	–	Cognitive + psychiatric	–	+	UL				–		GI symptoms
4.F/38	Two weeks	Moderate	165	3	61	Cognitive + psychiatric	–	–	BL			BL	+	CMV IgM	GI symptoms
5.M/35	Nine weeks	Severe	N/A	9	140	Cognitive impairment	–	+	BL		UL		+		
6.M/33	Three weeks	extremely severe	205	3	109	Severe encephalopathy	Left hemiparesis	+		BL		BL	+	CMV IgM	
7.F/30	Two weeks	Moderate	220	4	99	Cognitive impairment	Left hemihypoesthesia	+	BL		–	–	–	Anti Streptolysin	

Note: *S – symptomatic, **A-S – A-symptomatic. *** complete triad – evidence for CNS, retinal and Vestibulocochlear involvement; UN – unilateral, bilateral, *Abbreviations*: *CMV* – cytomegalovirus, *GI* – gastrointestinal, *WBC* –white blood cells

Five patients fulfilled the criteria for definite SuS and two patients for probable SuS. Their mean age at presentation was 30 years (range 20-38 years). All cases were diagnosed during the summer-autumn seasons (July-October). One women was pregnant in her 7th gestational week. The mean duration from the onset of the symptoms to diagnosis was three weeks (2-9 weeks). Mean duration of follow-up was 24 months and one patient was lost to follow after 2 months. No patient had previous neurological disease. Based on the detailed personal history, no common demographic characteristics were found among patients. Toxic environmental exposure was not reported.

At clinical onset, the most common manifestations were CNS symptoms. Disease severity was determined according to the extent of CNS involvement; one patient was defined as extremely severe SuS, two patients as severe SuS, three patients as moderate SuS and one as mild SuS. All patients suffered from different severity of encephalopathy characterized by cognitive impairment (mainly deficits in executive function, language and memory) or confusion. Three patients had a psychiatric manifestation of depression and anxiety. Three patients presented with focal neurological signs; sensory disturbance (two patients) and severe hemiparesis (one patient). Five patients had severe migrainous or oppressive headache. Visual disturbances were described as flashing lights in one patient, and visual field defects in three patients. Vestibulocochlear involvement was the least common presentation; three patients suffered from acute sensorineural hearing loss, one of these patients also suffered from vertigo. Gastrointestinal (GI) symptoms, i.e. abdominal pain and diarrhea were reported in three patients.

### Diagnostic procedures

**Fig. 1:**
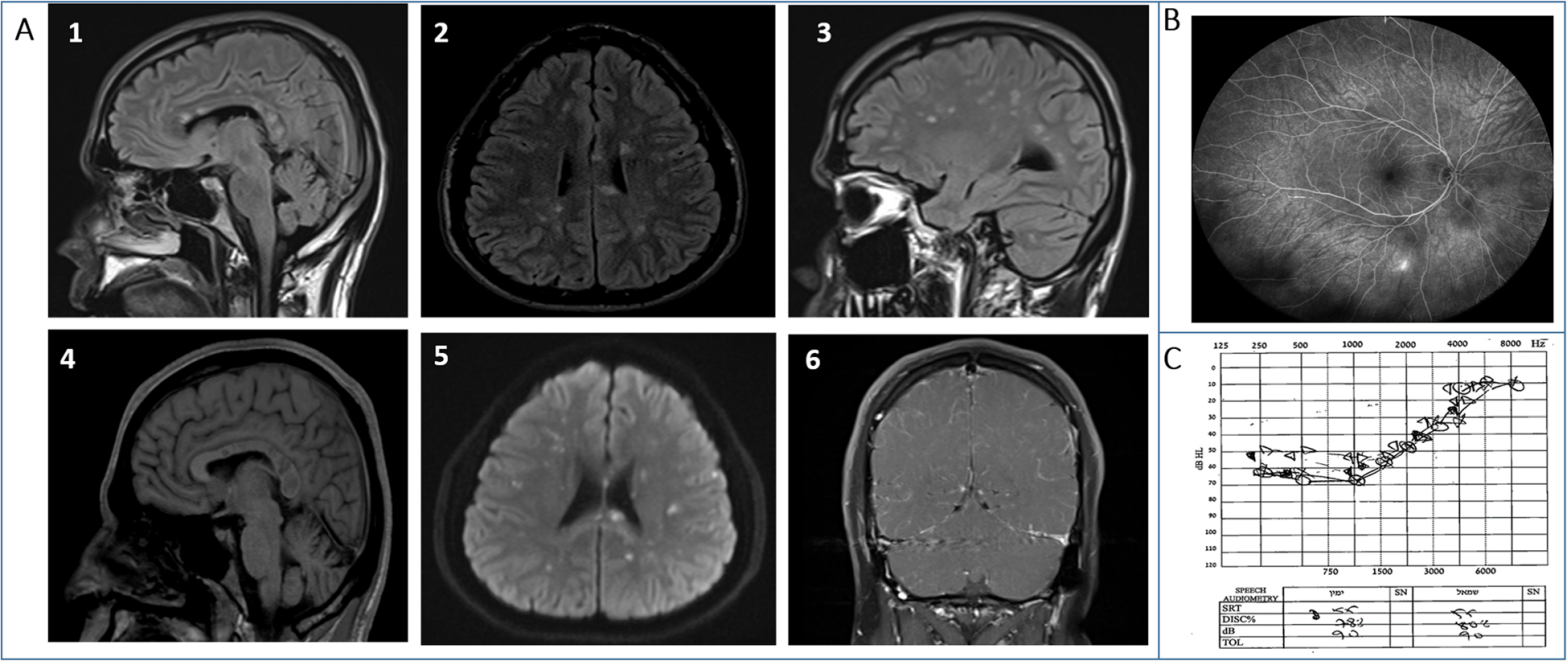
Typical diagnostic procedures findings. **a** Brain MRI. Sagittal and axial T2 FLAIR sequence, showing hyperintense lesions in the corpus callosum (“snow balls”). A_1_. Periventricular and subcortical areas. A_2-3_. Sagittal T1 sequence corpus callosum (“black holes”). A_4_. Axial DWI sequence which show restricted diffusion A5. Leptomeningeal enhancement with gadolinium A_6_. **b** Wide field Fluorescein angiogram demonstrating right eye peripheral (superior, temporal and inferior) branch artery occlusions and focal arterial leakage in an inferior branch artery. **c**. A bilateral low-tone and middle-tone sensorineural hearing loss is seen, with abnormal low scores in speech audiometry

Characteristic brain MRI findings included: 1. All patients presented with Flair/T2 hyperintense lesions located in the supratentorial white and gray matter areas. Three patients had additional infratentorial lesions. 2. Typical corpus callosum “snow ball” lesions that are considered a characteristic sign of SuS (Fig. 1a_1_) [21–23] were detected in all of our patients although was not evident on the initial imaging upon presentation in one patient. 3. Punctuate DWI hyperintense lesions with corresponding ADC hypointensity (restricted diffusion) were associated with disease activity in all of our patients (Fig. 1a) [9]. 4. Leptomeningeal enhancement was demonstrated in four patients (Fig. 1a_6_), three of these patients suffered from severe headache. 5. Corpus callosum hypointense T1 lesions were found in all our patients and corpus callosum atrophy was evident in five patients [24]. One of our patients (patient number 6) had a cervical spinal cord MRI done at disease onset. No evidence of cervical spine involvement was detected.

FA was pathological in all our patients, even though three patients had no ocular symptoms. Abnormalities included BRAOs and arterial wall hyperfluorescence (AWH) (Fig. 1b) [21, 22, 25]. One case had both arterial and venous occlusions. Audiometry showed low-frequency sensorineural hearing loss in five cases (Fig. 1c) [1, 6, 9, 18, 26], three were bilateral and two were unilateral. Of note, two patients had SNHL on audiometry with no auditory symptoms. All our patients performed OCT. Three patients showed signs of acute retinal hypoxia indicating an acute macular BRAO. Additionally, five patients showed signs of macular retinal thinning, compatible with previous events of macular ischemia. Two patients had normal OCT.

Serology for cytomegalovirus (CMV) was available in four patients. Three patients had IgM antibodies for CMV. CMV serology was retested in two patients; in one patient (Patient number one) there was sero-conversion from IgM to IgG and in the second patient (Patient number four) both IgG and IgM levels remained high during follow-up, this patient showed clinical and laboratory evidence of CMV reactivation. CMV PCR in the serum was done in two patients; patient number one had 543 IU/ml and patient number four had 70,635 IU/ml. CMV IgM antibody in the CSF were not measured. CMV immune-stain was done on tissue obtained from brain biopsy. One patient had high titer of anti-streptolysin antibodies. Anti-streptolysin titer in the CSF was not measured.

CSF analysis was performed in six patients (Table 1). Elevated protein levels were observed in all these patients (mean levels 107 mg/dl; 61- 140 mg/dl) and mean CSF cell count was 6 cells/μl (1-14 cells/μl). The presence of oligoclonal bands (OCB) in the CSF was examined in four patients and were all negative providing no evidence of intrathecal synthesis of OCB. Brain angiography was performed in two patients and was unremarkable. One of our patients (patient number 6) who presented with severe encephalopathy underwent stereotactic brain biopsy from an active lesion on his right parietal lobe. The samples include fragments of cortex, white matter and some vessels from subarachnoid space. There was no evidence of vasculitis or inflammatory reaction. CMV immune-stain was negative.

Figure 1 demonstrates typical diagnostic findings.

### Treatment regimen and clinical outcomes

Table 2 summarizes treatment regimens, major side effects and patient outcomes. Patients were treated according to disease course and the severity of CNS involvement based on the recently published treatment guidelines (Supplementary Table 2) [10]. IV Methyprednisolone (IVMP), intravenous immune globulin (IVIG) and mycophenolate mofetil were used for mild cases. An addition of Cyclophosphamide (CPM) and Rituximab (RTX) is optional for the treatment of moderate cases and is recommended to use in severe CNS involvement. All seven patients were treated with IV Methyprednisolone (IVMP) within the first month following the onset of symptoms and during relapses, followed by very slow tapering of prednisone. In addition, all patients received mycophenolate mofetil (MMF) or azathioprine. Six patients were treated with intravenous immune globulin (IVIG) 2 g/kg every 3-4 weeks or 1 g/mg every 2 weeks until clinical stability achieved. Cyclophosphamide (CPM; after fertility preservation) was administered in one patient with moderate SuS and in four patients with severe or extremely severe SuS. Three patients were treated with rituximab. Anti- aggregation is not included in the treatment guidelines and evidence for its efficiency in SuS in lacking [14, 15, 27], nevertheless, all seven patients were treated with an anti- aggregation agent.

**Table 2:** Treatment regimens, major side effects and patient outcomes

			Treatment						outcome			Side effects	Maintenece treatment
Patient	Disease Severity	Follwup time (months)	Anti aggregation drugs	IVMP	IVIG	CPM	MMF	RTX	Auditory	Vision	CNS		
1	Severe	33	Acetylsalicylic acid (+Clopidogrel)	+	+	+	+	+	Severe bilateral SNHL		No evidence for neurological residual	CMV reactivation	Aspirin, Prednisone, IVIG (1 g/kg), Rituximab,MMF
2	Moderate	33	Acetylsalicylic acid	+	–	+	+	–	SNHL	Bilateral VF deficient	No evidence for neurological residual		Aspirin,MMF
3	Mild	2	Acetylsalicylic acid	+	+	–	+	–	N/A	N/A	N/A	N/A	N/A
4	Moderate	30	Acetylsalicylic acid	+	+	–	+	+		Mild VF deficient	Fatigue	CMV reactivation	Aspirin, IVIG (1 g/kg), Rituximab
5	Severe	30	Acetylsalicylic acid	+	+	+	Azathioprine	+	SNHL	bilateral VF deficient	No evidence for neurological residual	Pathological fracture	Prednisone, Rituximab
6	extremely severe	21	Acetylsalicylic acid(+Enoxaparin)	+	+	+	+				Hemiparesis,ataxia and severe cognitive decline		Aspirin, Prednisone, IVIG (1 g/kg),MMF
7	Moderate	20	Acetylsalicylic acid	+	+	+	+	–			Mild cognitive decline		Aspirin, prednisone and IVIG (1 g/kg)

Note: N/A – not available; *Abbreviations*: *CMV* – cytomegalovirus, *CPM* – cyclophosphamid, *IVMP* – intravenous methylprednisolone, *IVIG* – intravenous immune globulin, *MMF* – mycophenolate mofetil, *RTX* - rituximab, *SNHL* - sensorineural hearing loss, *VF* – visual field

Patients were clinically monitored with frequent clinical assessments in addition to routine brain MRI, FA and audiometry to evaluate treatment response and outcome. Treatment led to clinical and radiological stability in all patients. One patient who presented with severe encephalopathy and hemiparesis was stable under this regimen but suffered from a residual mild hemiparesis, ataxia, memory impairment, attention deficit and behavioral disinhibition. Another patient experienced a relapse after discontinuation of medication due to adherence problem. This patient has a residual mild cognitive decline. Three patients had no residual neurological deficits. Brain MRI lesions remained unchanged in five patients however, in one patient some lesions disappeared on follow-up MRI. Follow up MRI revealed global brain atrophy and corpus callosum atrophy in four patients.

Two patients had residual visual field defects. OCT at final visit was performed in patients and showed bilateral thinning of retinal layers in five patients. Three patients who presented with SNHL did not improve on follow up auditory testing. Major side effects of the treatment included pathological fracture in one patient and reactivation of CMV infection in two patients that caused GI symptoms and was diagnosed by testing CMV PCR in serum. Patient number 4 suffered from severe diarrhea and serum CMV PCR reached a maximum level of 70,635 IU/ml and return to negative after treatment with valcyclovir.

The maintenance treatment included aspirin, mycophenolate mofetil (MMF) 1000 mg BID, prednisone with very slowly tapering down, decreasing doses of IVIG and Rituximab every 6 months.

## Discussion

In this case series, we describe seven patients diagnosed with SuS over a time period of thirteen months, representing at least a 5.4 - fold increase in the annual incidence of SuS expected in comparison to a published registry and a 7-fold increase in the annual incidence expected in our medical center. The clinical presentation and phenotype of our cohort was consistent with previously reported cases of SuS [4, 6, 7, 16–18]. We didn’t find any common environmental or toxic exposure preceding the disease onset.

The etiology of SuS remains unknown. An autoimmune process leading to occlusion of micro vessels have been postulated [2]. Elevated serum levels of anti-endothelial cells antibodies are found in approximately 25% of the patients, suggestive of an antibody-mediated immunity process [28, 29]. Recently a CD8+ T-cell-mediated endotheliopathy has been shown to play a key role in SuS [5]. What triggers this immune response is currently unknown.

Based on review of the literature, an infectious trigger does not seem to play a major role since it was detected only in 19 out of 304, of published cases [6]. Given a predisposition of the disease onset to the summer-autumn months and the absence of a clear disease trigger in our cohort, we searched for a possible infectious trigger. Interestingly, at presentation three quarters of our patients, who were tested for CMV, had positive IgM antibodies in the sera, while PCR for CMV in CSF was negative. Two of these patients suffered from CMV reactivation that caused GI symptoms following immunosuppressive treatment. In another patient, there was evidence of a recent streptococcal infection with a high serum anti-streptolysin titer. The fact that despite strong immunosuppressive therapy there was marked improvement and no eruption of symptoms supports an inflammatory post or para-infectious pathophysiology. Accordantly, the use of anti-viral treatment does not seem to be indicated. One hypothesis regarding disease pathophysiology that supports a para-infectious mechanism is the presentation of viral antigen on the endothelium following viral infection [5].

Two conclusions might be derived from our observation: the importance of searching for an infectious trigger, which might shed some light on disease mechanism and risk factors. Additionally, we believe that it is highly recommended to screen for possible latent infections due to robust immune suppression used to effectively treat these patients.

There is a great variability in the clinical presentation of SuS and the complete triad is present in less than 20% of patients at presentation. As a result, misdiagnosis or delay in diagnosis and treatment are common [5–7]. Diagnostic procedures such as MRI, FA, OCT [19, 20] and audiometry are crucial to enable early and accurate diagnosis since, as mentioned above, subclinical pathology may occur without clinical manifestation [5, 7, 18]. Of note, only one patient in this cohort presented with the complete triad of neurological, auditory and visual symptoms. Using these diagnostic tools, we were able to establish the diagnosis of definite SuS at presentation in five patients and to reduce time to diagnosis from twenty-one weeks to three weeks [6]. Moreover, close monitoring using these tools may allow the detection of silent disease activity. Anti-endothelial cell antibodies were not checked. Although high levels of anti-endothelial cell antibodies have been reported, titers > 1:100 were found in only 25% of patients with SuS and therefore, are not included in the diagnosis criteria of SuS [12].

GI symptoms reported by three of our patients may reflect the systemic nature of the disease as had already been proposed [30]. This could also be a result of preceding infection with CMV as two of these patients were CMV IgM positive.

Treatment of SuS is particularly challenging [10, 11, 22]. Importantly, Multiple sclerosis treatments may cause exacerbation of SuS [31]. Due to the rarity of the disease, no randomized controlled trials have been done. Rennebohm et al. published treatment guidelines based on large cohort of patients [10]. Treatment regimen is based on disease manifestation and severity. This is the first case series published using these treatment guidelines and it demonstrates that in order to achieve optimal outcomes, rapid and sometimes aggressive immunosuppressive combination therapy is required.

This study has several limitations. This is a retrospective case series that relies on medical records. There was some missing data, such as CMV status in three patients, serology follow-up of CMV levels, CMV serology in the CSF and antibody detection rate of IgM CMV antibodies in a control group. The small sample size makes it difficult to establish causal interference between infections and SuS, so that we cannot rule out the possibility of co-incidence or false positivity. It is important to recognize that potentially rise in awareness due recently published diagnostic criteria and suggested therapeutic guidelines [10, 18] led to earlier and more frequent detection of the syndrome. However, this publications did not cause a shift in diagnosis as criteria have not been revised.

Moreover, the mean follow-up time was less than two years, so that long-term outcomes cannot be drawn from this study. As this is a tertiary referral center, our case series probably includes more severe forms of SuS.

## Conclusion

Our case series adds to the present knowledge regarding this rare disorder. It emphasizes the variability in clinical presentation and highlights the importance of diagnostic procedures such as FA, audiometry and MRI in order to establish early diagnosis and avoid irreversible neurological damage. Further research on the pathophysiology of SuS and randomized controlled clinical trials are warranted to enable the development of evidence-based management strategies for these patients.

## Supplementary information

**Additional file 1 Supplementary figure 1.** PPT. Incidence of Susac syndrome between the years 2013-2018 in Tel-Aviv Medical Center. Each line represents a new diagnosis of Susac syndrome.

**Additional file 2 Supplementary Table 1.** DOC. Diagnostic criteria for Susac syndrome. Adopted from Kleffner et al. *J Neurol Neurosurg Psychiatry* 2016. Abbreviations: AWH = arterial wall hyperfluorescence; BRAO = branch retinal artery occlusion; FLAIR = fluid-attenuated inversion recovery; SD-OCT = spectral domain optical coherence tomography; SuS = Susac syndrome; SNHL = sensorineural hearing loss.

**Additional file 3 Supplementary Table 2.** DOC. Treatment guidelines according to severity of CNS involvement. Adopted from Rennebohm et al. *International journal of stroke* 2017. Abbreviations: BID twice a day; CPM-cyclophosphamid; IVIG – intravenous immune globulin; IVMP – intravenous methylprednisolone; MMF – mycophenolate mofetil; TAC-tacrolimus.

## Data Availability

The datasets used and/or analyzed during the current study are available from the corresponding author on reasonable request.

## Abbreviations

AWH: Arterial wall hyperfluorescence
BRAO: Branch retinal artery occlusion
CSF: Cerebrospinal fluid
CMV: Cytomegalovirus
CPM: Cyclophosphamide
CC: Corpus Callosum
FA: Fluorescein angiography
GI: Gastrointestinal
IVIG: Intravenous immune globulin
IVMP: IV Methyprednisolone
MMF: Mycophenolate mofetil
OBC: Oligoclonal bands
OCT: Optical coherence tomography
SuS: Susac syndrome
SNHL: Sensorineural hearing loss
RTX: Rituximab
