# Supplementary figures and images for: Increased incidence of Susac syndrome: a case series study
Source: BMC Neurol. 2020 Sep 2;20:332. doi: 10.1186/s12883-020-01892-0 (PMC7465403; doi:10.1186/s12883-020-01892-0)

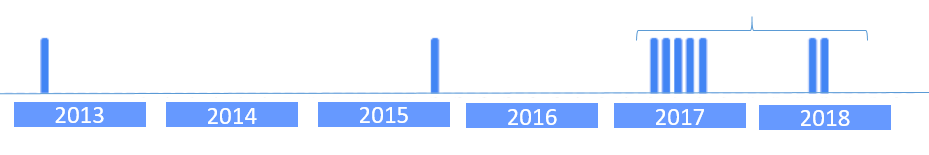

Supplement: Supplementary file 1 — Additional file 1 Supplementary figure 1. PPT. Incidence of Susac syndrome between the years 2013-2018 in Tel-Aviv Medical Center. Each line represents a new diagnosis of Susac syndrome. [file 12883_2020_1892_MOESM1_ESM.png]
