# Supplementary material for: Increased incidence of Susac syndrome: a case series study
Source: BMC Neurol. 2020 Sep 2;20:332. doi: 10.1186/s12883-020-01892-0 (PMC7465403; doi:10.1186/s12883-020-01892-0)
Supplement: Supplementary file 2 — Additional file 2 Supplementary Table 1. DOC. Diagnostic criteria for Susac syndrome. Adopted from Kleffner et al. J Neurol Neurosurg Psychiatry 2016. Abbreviations: AWH = arterial wall hyperfluorescence; BRAO = branch retinal artery occlusion; FLAIR = fluid-attenuated inversion recovery; SD-OCT = spectral domain optical coherence tomography; SuS = Susac syndrome; SNHL = sensorineural hearing loss. [file 12883_2020_1892_MOESM2_ESM.docx]

**Table 1: Diagnostic criteria for Susac syndrome**. Adopted from Kleffner et al. *J Neurol Neurosurg Psychiatry* 2016.
