# Supplementary material for: Increased incidence of Susac syndrome: a case series study
Source: BMC Neurol. 2020 Sep 2;20:332. doi: 10.1186/s12883-020-01892-0 (PMC7465403; doi:10.1186/s12883-020-01892-0)
Supplement: Supplementary file 3 — Additional file 3 Supplementary Table 2. DOC. Treatment guidelines according to severity of CNS involvement. Adopted from Rennebohm et al. International journal of stroke 2017. Abbreviations: BID twice a day; CPM-cyclophosphamid; IVIG – intravenous immune globulin; IVMP – intravenous methylprednisolone; MMF – mycophenolate mofetil; TAC-tacrolimus. [file 12883_2020_1892_MOESM3_ESM.docx]

**Table 2: Treatment guidelines according to severity of CNS involvement**. Adopted from Rennebohm et al. *International journal of stroke* 2017.

**Severity of CNS involvement**
